# Epidemiology of *Eustrongylides* sp. Infection in *Triplophysa strauchii*: Temporal Dynamics and Risk Factors

**DOI:** 10.3390/vetsci13070625

**Published:** 2026-06-26

**Authors:** Yuqing He, Monan Chen, Chaohao Yu, Xin Wang, Xinyang Li, Wei Guo

**Affiliations:** 1 Xinjiang Key Laboratory for Ecological Adaptation and Evolution of Extreme Environment Organisms, College of Life Sciences, Xinjiang Agricultural University, Urumqi 830000, China; hyq1218hyq@126.com (Y.H.);; 2 Postdoctoral Research Station of Animal Husbandry, Xinjiang Agricultural University, Urumqi 830052, China

**Keywords:** *Eustrongylides* sp., *Triplophysa strauchii*, epidemiology, parasitic infection

## Abstract

The epidemiology of the nematode *Eustrongylides* sp. remains poorly understood in natural fish populations, especially under alpine conditions. This study investigated its infection dynamics in *Triplophysa strauchii*, an endemic fish in an alpine wetland in northwestern China. A total of 720 fish were sampled monthly from April to November 2025. Parasitic larvae were identified using ribosomal ITS sequences. Hurdle models were employed to assess infection risk and intensity, incorporating host length, sex, and month as predictors. The overall prevalence was 21.8%, with a mean intensity of 4.36 ± 6.83 worms per infected fish. Infection dynamics showed two seasonal peaks: a spring peak with high intensity and a late-summer peak with moderate prevalence but low intensity. Host length was the most stable predictor for both infection risk (OR = 1.916 per cm, 95% CI: 1.232–2.977) and intensity, with significant length × month interactions (length × May: IRR = 4.847, *p* = 0.005; length × November: OR = 0.418, *p* = 0.005), indicating seasonal modulation of the length effect. These findings provide foundational insights into parasite transmission in alpine wetlands and highlight public health concerns regarding fish consumption.

## Simple Summary

*Eustrongylides* is a parasitic nematode whose life cycle involves fish as second intermediate hosts and piscivorous waterbirds as definitive hosts. In humans, the consumption of undercooked, infected fish can lead to gastrointestinal symptoms such as enteritis and diarrhea. This study reports, for the first time, the infection of a local fish called *Triplophysa strauchii* in Xinjiang, China. After checking 720 fish over eight months, we found that one in five carried the worms. Infections peaked in spring and late summer. Larger fish were more often infected and harboured more worms, and the effect of body length varied significantly across months. This new finding matters for food safety and fishery management in cold wetlands.

## 1. Introduction

Parasites are important components of ecosystems and influence host population dynamics and community structure [1,2,3]. Wetlands connect aquatic and terrestrial environments, and their hydrology and biodiversity can facilitate the maintenance and transmission of parasites [4]. Gaojiahu Wetland, located in Hami, Xinjiang, China, is a wetland in eastern Xinjiang with extensive open water that supports diverse bird and aquatic communities [5]. The water source of this wetland mainly relies on Tianshan Mountain ice and snow meltwater recharged through underground runoff, supplemented by atmospheric precipitation, forming a relatively unique and fragile hydrological process. The water area has been in a low-temperature environment for a long time, with a long ice-covered period from November to March, followed by ice melting in April. The water temperature shows strong seasonal fluctuations, and the growing season is extremely short. It has a temperate continental arid climate and has significant alpine characteristics [6].

The genus *Eustrongylides* (order Enoplida, family Dioctophymatidae) has a complex life cycle [7] that involves multiple developmental stages and various hosts. It is also widely distributed around the world [8,9]. Members of the genus parasitize a broad range of hosts, including birds, fish, reptiles, and amphibians. Migratory birds, as important vectors of transmission, can cross geographical barriers during migration, further increasing the risk of cross-regional transmission. Xia et al. [10] first reported this nematode from the population of the loach *T*. *microphthalma* in Da Caohu Lake, Turpan, Xinjiang, China. Through artificial infection experiments combined with molecular identification (phylogenetic analysis based on ITS sequences), the parasite was definitively identified as *Eustrongylides tubifex* [11], representing a new host record of this nematode species in China.

During a biological survey of *T. strauchii* in Xinjiang, we found that the population of this fish in the Hami area is also infected with nematodes. After morphological comparison, the nematode is similar to the nematode sample collected by our team in the waters of Dacao Lake in Turpan, and we initially suspected it belonged to the same genus. To determine the taxonomic affiliation (at least to genus) of this nematode and to characterize its infection dynamics, an eight-month monitoring of *T. strauchii* in the waters of Gaojia Lake was carried out. This study aimed to (i) identify the parasite using ITS sequences, (ii) characterize the seasonal infection dynamics of *Eustrongylides* sp. in *T. strauchii*, and (iii) assess the effects of host body length, sex, and month on infection risk and intensity, thereby offering a scientific basis for fish resource management and disease surveillance in arid-zone wetland ecosystems.

## 2. Materials and Methods

### 2.1. Study Location and Sampling

**Figure 1:**
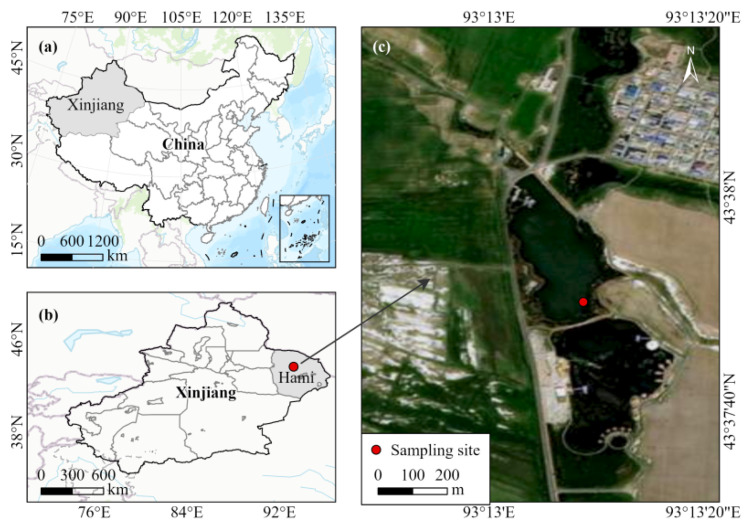
Geographic location of the sampling site in Gaojiahu Wetland, Hami, Xinjiang, China. (**a**) Schematic map of the Xinjiang Uygur Autonomous Region in China; (**b**) Schematic map of Hami City in the Xinjiang Uygur Autonomous Region; (**c**) Schematic map of the sampling site at Gaojiahu Wetland in Hami City.

This study was conducted from April to November 2025 in the waters of Gaojia Lake Wetland, Hami region, Xinjiang, China (Figure 1; approximately 43.63° N, 93.22° E). Monthly sampling was performed using stationary gill nets (mesh size: 1 cm, net length: 3 m, soak time: 12 h) deployed at three fixed sites within the wetland (water depth: 0.5–1.0 m), collecting a total of 720 individuals of *T. strauchii* (90 individuals randomly selected per month). Captured live fish were immediately placed in aerated water tanks and rapidly transported to the laboratory for subsequent processing.

### 2.2. Host Biometric Measurements

Fish were anesthetized using MS-222 (tricaine methanesulfonate) at a concentration of 100 mg/L buffered with sodium bicarbonate for 3–5 min until opercular movements ceased. After biometric measurements, fish were euthanized by an overdose of anaesthetic (200 mg/L MS-222) followed by pithing. Subsequently, the standard length of each fish was measured using an electronic digital caliper (precision 0.01 cm), and body weight was measured using an electronic balance (accuracy 0.01 g). The fish were then dissected, and their sex was determined based on the external morphology of the gonads (males had paired whitish testes, females had yellowish ovaries; individuals with no distinguishable gonads were recorded as unknown).

### 2.3. Parasite Inspection and Preliminary Identification

After systematic dissection, the mesentery, body cavity, surfaces of internal organs, and the skeletal muscle were examined for red, free-living nematode larvae and worms enclosed in white cysts. Preliminary morphological observations and counts of all worms were conducted under a stereomicroscope, and the number of parasites in each fish was recorded. Based on the morphological characteristics of the nematode body (such as body length, head-end structure, tail shape), it was initially identified as *Eustrongylides*. The worm specimens were rinsed with 0.9% saline solution, fixed in 95% ethanol, and stored at −20 °C for subsequent molecular identification.

### 2.4. Molecular Identification of Parasites

Genomic DNA was extracted from individual worms using a commercial extraction kit (OMEGA Bio-Tek, Norcross, GA, USA), the internal transcribed spacer (ITS) region was amplified by PCR using primers 18S-F and 28S-R (Table 1), and PCR products were purified and bidirectionally sequenced by Sangon Biotech (Shanghai) Co., Ltd. (Shanghai, China). All primers were synthesised by Sangon Biotech. The obtained ITS sequences were subjected to BLAST homology alignment in the NCBI GenBank database.

### 2.5. Data Processing and Statistical Analysis

The following infection indices were calculated according to the definitions of Margolis et al. [12] and Bush et al. [13]: prevalence (number of infected individuals/total number examined × 100%), mean abundance (total number of parasites/total number examined, including zeros), and mean intensity (total number of parasites/number of infected individuals, i.e., among infected fish only), along with their standard deviations.

All statistical analyses were performed in R 4.4.1. The length–weight relationship was fitted using the log-linear model ln(W) = ln(a) + b ln(L). A *t*-test was used to test whether b equals 3 (isometric growth). Monthly b values were obtained by fitting the model separately for each month. A binomial logistic regression was fitted with infection status as the dependent variable and body length (L), sex, and month as independent variables. **No variable selection was performed**; the full model is reported.

For infection intensity (zero-inflated count data), a hurdle negative binomial model was employed, consisting of a binary component (logit link for infection probability) and a count component (log link for intensity among infected fish). Two candidate models were compared: a main-effects model (L + month + sex) and a model including the L × month interaction. The interaction model had a slightly lower AIC (1337.44 vs. 1340.02, ΔAIC = 2.57) and produced no negative estimated zero probabilities after correct inverse logit transformation; therefore, the interaction model was retained for its superior fit and interpretability. Model fitting was performed using the pscl package in R with the hurdle() function. Zero probabilities were calculated manually as P(zero) = 1 − plogis(X_zero %*% β_zero), where X_zero is the model matrix for the zero hurdle component and β_zero is the corresponding coefficient vector. This approach ensures all predictions lie within (0, 1). The final model was then used to obtain odds ratios (OR) and incidence rate ratios (IRR). Model diagnostics included the dispersion parameter θ, the ratio of Pearson χ^2^ to residual degrees of freedom, and a comparison of observed versus predicted zero proportions. No prediction accuracy based on a 0.5 threshold was reported because the model was not intended for prediction. The relative condition factor (Krel) was not included in the final analysis because it was not significant in preliminary tests and its inclusion would cause collinearity with body length.

The significance level was set at α = 0.05. Raw data and analysis code are available from the corresponding author upon reasonable request.

### 2.6. Ethics Statement

This experiment complied with laboratory animal ethics and wildlife sampling permits, and all procedures complied with relevant laws. The study was certified by the Institutional Animal Care and Use Committee (permit number: 2024049).

## 3. Results

### 3.1. Molecular Identification Based on ITS Sequences

The ITS regions were amplified by PCR, and the resulting sequences (a representative sequence deposited in GenBank under accession number PZ532769) were analyzed using BLAST on NCBI. The results showed that the sequence submitted by Xia et al. (accession number PQ519610) exhibited the highest similarity to the sequences obtained in this study, with 98.76% identity. Further genetic distance analysis revealed that the nematode was most closely related to *Eustrongylides tubifex* (genetic distance 0.0091), followed by *Eustrongylides ignotus* (0.0155) and *Eustrongylides excisus* (0.2113) (Table 2). This indicates that the parasite detected in this study belongs to the genus *Eustrongylides*. However, due to the absence of adult morphological identification, it could not be identified to the species level. Therefore, the parasite is referred to as *Eustrongylides* sp. throughout this paper.

### 3.2. Infection Status of T. strauchii with Eustrongylides sp.

**Figure 2:**
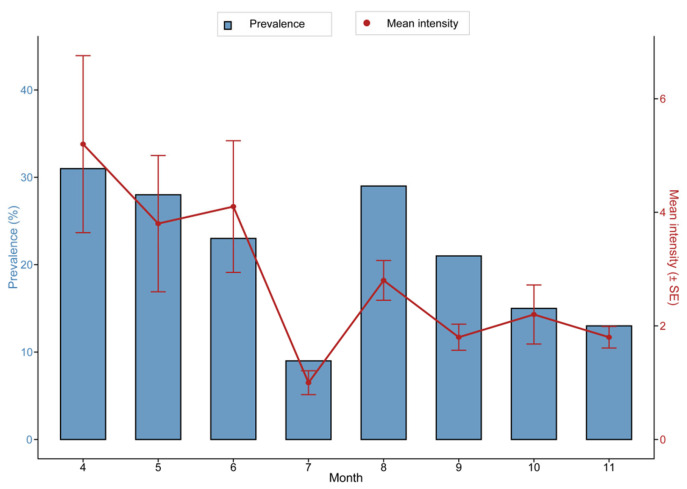
Monthly variation in prevalence and mean intensity of *Eustrongylides* sp. in *T. strauchii* (error bars represent ± SD).

From April to November 2025, a total of 720 *T. strauchii* individuals were collected. The overall prevalence of *Eustrongylides* sp. was 21.81% (Table 3). The mean intensity was (4.36 ± 6.83) worms per infected fish (range: 1–43). Infection parameters exhibited marked seasonal fluctuations (Figure 2). Prevalence peaked in April at 31.1%, reached its lowest point in July at 10.0%, and then increased again in August to 28.9%. Mean abundance followed a similar trend, being highest in April (3.03 ± 7.64) and lowest in July (0.12 ± 0.39). The mean intensity of infection among parasitized fish was highest in April (9.75 ± 8.24), then fluctuated and generally decreased, reaching its lowest value in November (1.47 ± 0.72).

### 3.3. Allometric Relationship Between Body Length and Body Weight of T. strauchii

**Figure 3:**
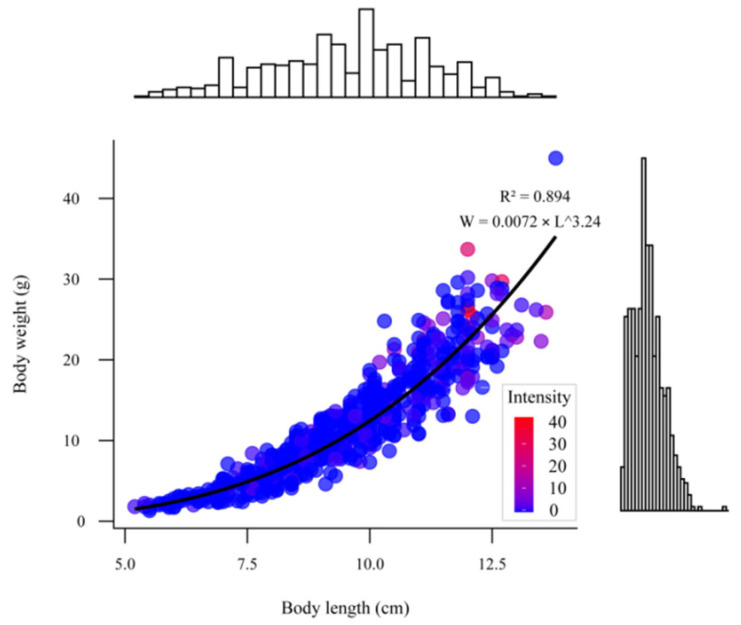
Relationship between body length and body weight of *T. strauchii,* with infection intensity indicated by color. Note: Relationship between body length and body weight of *T. strauchii*. Points are colored by infection intensity (blue: low, red: high). The black curve represents the global allometric growth model: W = 0.0072 × L^3.24^ (R^2^ = 0.894). Marginal histograms show the distributions of length (top) and weight (right).

The global allometric model was ln(W) = –4.94 + 3.24 ln(L) (R^2^ = 0.894, F_1718_ = 6035, *p* < 0.001), i.e., W = 0.0072 × L^3.244^ (Figure 3). The isometry test showed that the overall b value was significantly greater than 3 (t = 5.84, *p* < 0.001), indicating positive allometric growth in the fish. Monthly fitted b values are presented in Table 4. Positive allometry (b > 3) was observed in May (3.61), October (3.29), and November (3.50) (all *p* < 0.01). For September, b = 2.90 (*p* = 0.374), indicating isometric growth rather than negative allometry. For the remaining months, growth was isometric (*p* > 0.05).

### 3.4. Hurdle Negative Binomial Model for Infection Intensity

Two candidate hurdle models were compared: a main-effects model (L + month + sex) and a model including the L × month interaction. The interaction model had a lower AIC (1337.44 vs. 1340.02, ΔAIC = 2.57) and produced no negative estimated zero probabilities after correct inverse logit transformation. Therefore, the interaction model was retained. The final model parameters are shown in Table 5.

Zero-hurdle part (infection probability): Body length had a significant positive effect (OR = 1.916, 95% CI: 1.232–2.977, *p* = 0.004). The length × November interaction was significant (OR = 0.418, 95% CI: 0.228–0.766, *p* = 0.005), indicating that the positive effect of body length on infection risk was weaker in November. Other monthly interactions and sex were not significant.

Count part (infection intensity among infected fish): The length × May interaction was significant (IRR = 4.847, 95% CI: 1.612–14.577, *p* = 0.005), indicating that the positive effect of body length on infection intensity was amplified in May. Month main effects (vs. April) were significant for most months, but length was not significant as a main effect in the count part after including interactions. Sex was not significant in either part.

### 3.5. Model Diagnostics

In this study, the negative binomial dispersion parameter θ was 0.296, indicating overdispersion. The ratio of Pearson χ^2^ to residual degrees of freedom was 1.054, suggesting no lack of fit. The observed proportion of zero counts was 78.19%, and the predicted proportion (calculated manually via inverse logit transformation of the zero-hurdle component) was 78.19%, indicating perfect correspondence between observed and predicted zero inflation. A residual plot (Figure S1) showed no systematic pattern.

### 3.6. Correlation Between Monthly Allometric Exponent b and Infection Parameters

**Figure 4:**
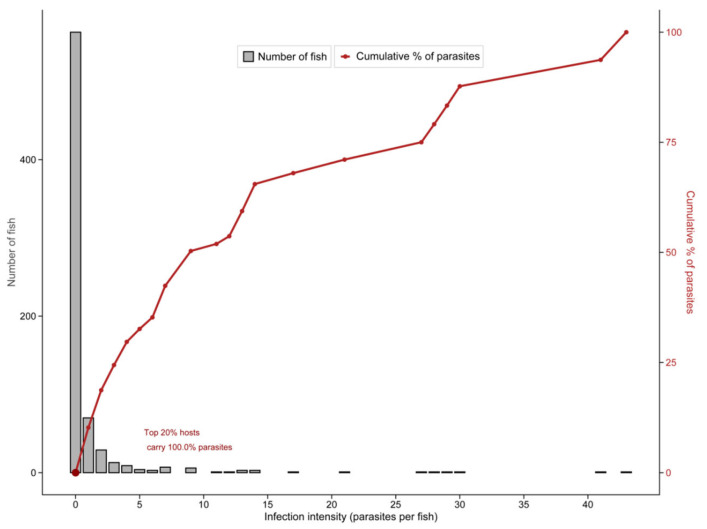
Aggregated distribution of *Eustrongylides* sp. in the host population (*T. strauchii*). Note: Aggregated distribution of *Eustrongylides* sp. in the *T. strauchii* population. Gray bars represent the number of fish at each infection intensity (left *y*-axis). The red line and points show the cumulative percentage of parasites (right *y*-axis). The red dot indicates that, among infected fish, the most heavily infected 20% harboured a disproportionately large fraction of the total parasite load (calculated as 67.5%).

The monthly allometric exponent b showed no significant correlation with either prevalence (r = −0.038, t = −0.09, df = 6, *p* = 0.93) or mean abundance (r = 0.013, t = 0.03, df = 6, *p* = 0.98) based on eight monthly observations (n = 8), which provides low statistical power, indicating that no significant association was detected between growth pattern and parasite infection (Figure 4).

## 4. Discussion

The internal transcribed spacer (ITS) of ribosomal DNA is a widely used molecular marker for the taxonomic identification of parasites [14], and has been extensively applied in molecular phylogenetic studies of the genus *Eustrongylides* [15,16]. Currently, two named species, E. tubifex and E. ignotus, are globally recognised, together with an unidentified *Eustrongylides* sp. [11]. Based on the ITS sequence molecular identification, the nematode obtained in this study was placed in the genus *Eustrongylides* and showed a high genetic similarity to *E. tubifex*. However, the sequence information for valid species of this genus is limited, and no clear interspecific genetic distance threshold has yet been established for molecular identification [17]. In addition, the larvae of *Eustrongylides* species are extremely similar in morphology, and precise species-level identification still depends on the morphological features of adults. Consequently, the specimen in this study is provisionally identified as *Eustrongylides* sp.

Species of *Eustrongylides* are nematode parasites with an extremely complex life cycle, and their life cycle involves a variety of hosts such as fish and birds [18,19,20]. Although relevant reports exist both domestically and internationally [8,21,22,23,24], the records of their intermediate and definitive hosts are not comprehensive [25].

This study reports the first occurrence of *Eustrongylides* sp. from the native fish *T. strauchii* in Xinjiang. By integrating molecular identification, seasonal sampling, and Hurdle model analysis, a systematic epidemiological investigation was conducted. The results showed that the parasite was *Eustrongylides* sp., and its infection dynamics showed seasonal fluctuations with a main peak in spring and a secondary peak in late summer. It was determined that host body length was a stable and significant intrinsic factor that affected the risk of infection, with its effect significantly modulated by month (length × month interactions). These patterns provide new insights into how environmental seasonal changes may shape parasitic relationships in alpine wetland ecosystems, although direct measurements are needed to confirm the underlying mechanisms.

### 4.1. Life Cycle of Eustrongylides spp.

*Eustrongylides* sp. is a globally distributed parasite with a complex life cycle involving two intermediate hosts and one definitive host. Aquatic oligochaetes serve as the first intermediate host [19]; fish (especially plankton-feeding and benthic fish) act as the second intermediate host, ingesting the infected larvae; the definitive host is piscivorous birds, with adult worms mainly parasitizing the stomach of waterbirds [18,20]. The larval stage mainly parasitizes fish, and after development, the parasite enters birds through predation.

Current knowledge regarding the intermediate and definitive hosts of this parasite remains incomplete [25]. In China, Moravec et al. [26] reported a new host record of *Eustrongylides* in *Paramisgurnus dabryanus* from Hubei Province; Xia et al. [10] also recorded this parasite in *T. microphthalma* from the Turpan region of Xinjiang. Internationally, infections of fish, birds, and even Nile crocodiles have been reported in Italy [21], Brazil [8], and Peru [22,23,24]. Thus, *Eustrongylides* is widely distributed around the world, and infections in aquatic animals and poultry have been documented in multiple regions of China.

Xinjiang is located along the important Central Asian migratory bird flyway, where a large number of waterbirds stop over and then continue their migration [27,28]. Accordingly, it is hypothesized that some waterbirds may have been infected with *Eustrongylides* while feeding in other areas. After migrating to Xinjiang waters, they release parasite eggs through defecation, contaminating the local water bodies. Aquatic oligochaetes ingest the eggs, and the larvae develop and infect native fish, including *T. strauchii*. This study reports the first collection of *Eustrongylides* from *T. strauchii*, providing a new addition to the research on the intermediate hosts of this nematode.

### 4.2. Seasonal Infection Dynamics: Spring Peak and Late-Summer Resurgence

Located at the foot of the East Tianshan Mountains, the waters of Gaojia Lake have an average altitude of 1600 m. It has a typical temperate continental climate, with long, cold winters, with a five-month ice-covered period (from November to March), and ice melt typically occurs in April. This environmental feature constitutes strong natural selection pressure, prompting the aquatic organisms living here to evolve special survival strategies, and has an important impact on the species composition and biological adaptability of the lake ecosystem. The infection situation of fish-parasitic nematodes has obvious seasonal patterns and is closely related to their growth and development cycle. Water temperature also directly affects the development and life history of parasites, thereby affecting the intensity of parasite infection [29]. Seasonal fluctuations in parasite infection burden have been widely documented in a variety of host–parasite systems [30], with significant differences observed between months [30,31].

In this study, the infection dynamics of *T. strauchii* infected with *Eustrongylides* sp. also showed distinct seasonal characteristics.

The long ice-covered period may create a distinct seasonal pattern by suppressing foraging and intermediate host populations during winter; after ice melt in April, a “resource pulse” of zooplankton and benthic invertebrates may coincide with peak host feeding, leading to high infection [32,33]. This pattern is consistent with the hypothesis of environmentally mediated changes in host exposure [30]: seasonal changes drive infection dynamics by affecting the probability of host exposure to infectious stages, but direct measurements of water temperature, intermediate host abundance, and fish diet are needed to confirm this mechanism. The infection experiments of *Eustrongylides* sp. in mallards conducted by Fastzkie et al. [34] and the observations in Japanese little grebes (*Tachybaptus ruficollis*) by Asakawa et al. [35] both support that the infection dynamics of this parasite fluctuate with seasonal changes, which is consistent with the spring peak observed in this study.

Entering summer, infection dynamics experienced a sharp reversal. The increase in water temperature in summer may directly lead to the death or expulsion of larval parasites in fish, or may reduce the survival of free-living stages [31]. However, the potential impact of summer high temperatures on parasite transmission in our study area remains speculative and requires direct investigation.

Shearer and Ezenwa [36] found, in their study on Grant’s gazelles in East Africa, that environmental changes (rainfall) not only affect host susceptibility, but also cause delayed effects on host infection with parasites. In the waters of Gaojia Lake, a similar delayed effect could occur, and high temperatures in summer might also directly affect parasite survival. In addition, the life cycle of the parasite itself cannot be ignored. Fastzkie et al. [34] observed that the larvae of *Eustrongylides* sp. in fish have a limited lifespan and a reduced survival rate at high temperatures, which also supports the view that a large number of larvae die in summer, leading to a decline in infection load. The sharp decline in infection parameters in July could arise from one or more factors, such as temperature-induced parasite mortality, selective removal of heavily infected hosts, or a seasonal change in fish diet. Further studies are needed to evaluate these possibilities.

It is worth noting that the sub-peak that occurred in August was characterized by “moderate prevalence (28.9%) with low intensity”, and its intensity was significantly lower than that in spring. According to the Hurdle model, infection intensity in August (2.54 ± 1.79) was significantly lower than in April (9.75 ± 8.24) (IRR = 0.16, *p* < 0.001; Table 5). This pattern suggests that after summer, the host population begins to be exposed to the source of infection again, but due to the short accumulation time, the number of parasites carried by each fish is limited. Therefore, the rebound in August was not a simple repeat of the spring pattern, but could represent a restart of the infection process. The “seasonal reproduction—susceptible host recruitment” mechanism proposed by Altizer et al. [30] provides a robust explanation for this phenomenon. After summer, juvenile fish join the population, which means that a large number of juvenile fish (which may be immunologically naive) enter the population, causing the infection rate to rebound rapidly. As the accumulation time is still short, the number of parasites carried by each fish is limited, so the intensity is still low. Alternatively, acquired immunity or short exposure time could limit parasite accumulation, but these were not measured.

In summary, the environmental gradient shaped by the ice-covered period in Gaojia Lake may contribute to the unique annual fluctuation pattern—from spring peak to summer decline to autumn sub-peak—by modulating host exposure windows, parasite survival, and the recruitment of susceptible hosts. However, water temperature, oligochaete density, and bird abundance were not measured in this study; therefore, the above mechanistic interpretations remain hypothetical and await further validation with independent data.

### 4.3. Body Length as a Stable Predictor of Infection Risk

In natural waters, fish are often exposed to multiple environmental stresses at the same time, including temperature fluctuations, water quality changes, pollutant exposure, and parasitic infections. Under the combined effect of these multiple pressures, the interaction between parasites and hosts becomes more complex [37]. Gaojia Lake is a typical plateau natural water area, and its seasonal temperature fluctuations and dissolved oxygen changes constitute continuous environmental pressures. These pressures may cause differences in the resistance of individuals of different body lengths to nematodes by regulating the immune status of the host, thus manifesting as a correlation between body length and infection intensity.

According to the hurdle model, body length emerged as a stable and significant predictor of infection risk throughout the study period (OR = 1.916, *p* = 0.004), establishing host size as a key factor governing infection probability. However, the effect of body length was not uniform across months: the length × November interaction (OR = 0.418, *p* = 0.005) indicated that the positive effect of body length on infection risk was significantly weaker in November, while the length × May interaction (IRR = 4.847, *p* = 0.005) showed that the effect of length on infection intensity was amplified in May. This seasonal modulation suggests that ontogeny interacts with environmental conditions to determine infection burden.

Analysis of growth parameters provides a host-level physiological basis for understanding infection dynamics. In this study, the monthly fitted b values (Table 3) showed that *T. strauchii* exhibited overall positive allometric growth (b = 3.24 > 3) (Figure 3), but its growth followed a clear seasonal rhythm: positive allometric growth occurred in May, October, and November (b > 3, *p* < 0.01); the b value in June was marginally lower than 3 (*p* = 0.052), approaching negative allometric growth; in the remaining months, growth was isometric (*p* > 0.05), indicating a relatively stable growth pattern.

A comparison between growth rhythm and the seasonal infection dynamics (Figure 2) revealed important associations. The peak infection levels in April (prevalence 31.1%; intensity 9.75) coincided with the breeding peak of a congeneric species, *Triplophysa yarkandensis* [38], suggesting a possibly similar reproductive timing in *T. strauchii* and the post-thaw emergence of aquatic oligochaetes, the first intermediate hosts [19]. Breeding-related aggregation and physiological stress [39,40], combined with the “resource pulse” of intermediate hosts, might have driven the high infection risk in spring. Positive allometric growth (b > 3, *p* < 0.01) occurred in May, October, and November, reflecting periods of active feeding and energy accumulation. However, infection outcomes during these months diverged markedly (Figure 2): May maintained high prevalence (26.7%) and intensity (4.83), whereas October–November exhibited low prevalence (15–17%) and intensity (1–2). This indicates that active feeding is necessary but not sufficient for high infection. In May, breeding-enhanced susceptibility and intermediate host availability may have amplified transmission. In autumn, despite active feeding, reduced water temperatures likely suppressed intermediate host populations or the survival of free-living parasite stages, limiting transmission. By June, post-breeding fish exhibited near-negative allometry and reduced feeding, leading to fewer new infections and paving the way for the July trough (prevalence 10.0%; intensity 1.22). The stable relationship between body length and infection also provides an important explanation for understanding the population distribution pattern of parasites. This study found that *Eustrongylides* sp. had a typical aggregated distribution in the *T. strauchii* population (Figure 4), i.e., a few fish harboured many nematodes, while most individuals harboured very few or none. As the most common distribution type of parasite populations, the formation of clustered distribution is related to various factors such as differences in host sensitivity, the ability of parasites to accumulate in the host (since *Eustrongylides* larvae do not reproduce in fish), and the host’s immune clearance ability [41]. It is plausible that larger individuals continue to accumulate more parasites, thereby exacerbating the uneven distribution. Lafferty and Kuris [42] suggested that environmental stress modulates parasite traits such as body size and virulence, contributing to geographic variation in parasite characteristics. In line with this, the robust length–infection association observed in our study represents one manifestation of the complex interplay between environmental stressors, host physiological status, and parasite adaptation.

### 4.4. Implications for Public Health and Ecosystem Management

*Eustrongylides* sp. is regarded as a zoonotic parasite of potential public health concern [7]. Its pathogenic effects on fish include nutrient exploitation [43], mechanical damage, and secondary infections [44]. Humans can become infected by consuming undercooked fish containing infective larvae, leading to eustrongylidosis, which manifests as enteritis, diarrhea, and other gastrointestinal symptoms [45].

The present study documents the occurrence of *Eustrongylides* sp. in *T. strauchii*, thereby expanding the known intermediate host spectrum for this parasite. *T. strauchii* is a common native fish species in plateau regions and is traditionally harvested and consumed by local residents. Although no human cases of *Eustrongylides* sp. infection have been reported in China to date. Nevertheless, consumers should be advised to thoroughly cook fish and avoid raw consumption. Its presence in fish highlights a potential food safety risk. The high infection intensity recorded in April (mean: 9.75) is particularly concerning as this period coincides with the post-ice-melt fishing season. Di Maggio et al. [7] also detected *Eustrongylides* spp. in fish from lakes in central Italy and emphasized the associated risks to local fishery supply chains. Furthermore, a five-year monitoring study by Franceschini et al. [46] on Lake Trasimeno fishery confirmed that the persistent occurrence of *Eustrongylides* spp. necessitates strengthened risk management.

In addition, the transmission of *Eustrongylides* sp. is closely associated with water pollution and waterbird migration [47]. Xinjiang lies along a major Central Asian migratory flyway, where large numbers of waterbirds stop over during migration [27,28], potentially introducing *Eustrongylides* spp. into local waters and infecting native fish. Zaharieva et al. [47], who detected *Eustrongylides* spp. in gobies from the Danube River, also considered bird migration a key transmission factor. Similarly, Shen [48] reported *Eustrongylides mergorum* in the Chinese merganser (*Mergus squamatus*) from Weishan Lake, Shandong Province, further corroborating the critical role of birds in the dissemination of *Eustrongylides*. Therefore, it is recommended to enhance surveillance of parasites in wild fish populations in Xinjiang, assess the prevalence and associated risk of *Eustrongylides*, and ensure the safety and quality of aquatic products.

Among *T. strauchii*, larger individuals carried the highest parasite loads. This information can be used to identify high-risk groups and guide the development of monitoring and sampling schemes.

The hurdle model accurately predicted zero counts (78.19% observed vs. 78.19% predicted after correction of prediction extraction), indicating that the included host factors (length, month, sex, and their interactions) explain the zero-inflation pattern well. The previously reported discrepancy was due to incorrect prediction extraction. Future studies should nevertheless incorporate environmental covariates (e.g., water temperature, oligochaete abundance, bird density) to further improve model performance.

## 5. Conclusions

This study provides the first report of *Eustrongylides* sp. in *T. strauchii* from an alpine wetland in Xinjiang. Infection showed monthly variation during April–November, with highest prevalence and intensity in April and a secondary peak of moderate prevalence in August. Host body length was the most consistent predictor of infection risk (OR = 1.916 per cm, 95% CI: 1.232–2.977), but its effect was significantly modulated by month: the length–November interaction weakened the positive length effect on infection risk (OR = 0.418), while the length–May interaction amplified the length effect on infection intensity (IRR = 4.847). Sex had no detectable effect. The seasonal patterns suggest a decline of infection over summer, but the underlying mechanisms (e.g., temperature effects, intermediate host dynamics) remain to be directly tested. These findings advance our understanding of parasite transmission in highland aquatic ecosystems and offer scientific support for fish resource management and food safety evaluation in the area.

## Figures and Tables

**Table 1:** Primers and reaction conditions.

Gene	Primer (5′-3′)	Reaction Condition
*ITS*	18S-F (5′-TTGGATGATTCGGTGAGGT-3′)	Initial denaturation at 94 °C for 5 min; 35 cycles of denaturation at 94 °C for 30 s, annealing at 55 °C for 30 s, extension at 72 °C for 30 s; final extension at 72 °C for 8 min, hold at 4 °C.
	28S-R (5′-AACCGCTTAGTAATATGCT-3′)	

**Table 2:** Genetic distance analysis of *Eustrongylides*.

Species Name	*Eustrongylides* sp.	*E*. *tubifex*	*E. ignotus*	*E*. *excisus*
*Eustrongylides* sp.	-	-	-	-
*E*. *tubifex*	0.0091	-	-	-
*E*. *ignotus*	0.0155	0.0128	-	-
*E*. *excisus*	0.2113	0.2105	0.2109	-

**Table 3:** Monthly infection parameters of *Eustrongylides* sp. in *T. strauchii* from April to November 2025.

Month	Sample Size	Number Infected	Prevalence (%)	Mean Abundance	Mean Intensity
4	90	28	31.1	3.03 ± 7.64	9.75 ± 8.24
5	90	24	26.7	1.29 ± 4.85	4.83 ± 5.90
6	90	22	24.4	1.53 ± 4.00	6.27 ± 5.43
7	90	9	10.0	0.12 ± 0.39	1.22 ± 0.63
8	90	26	28.9	0.73 ± 1.49	2.54 ± 1.79
9	90	19	21.1	0.32 ± 0.78	1.53 ± 1.02
10	90	14	15.6	0.32 ± 0.98	2.07 ± 1.94
11	90	15	16.7	0.24 ± 0.61	1.47 ± 0.72
Total	720	157	21.81	0.95 ± 3.67	4.36 ± 6.83

**Table 4:** Monthly allometric factor b and its comparison with 3.

Month	b ± SE	95% CI	t (vs. 3)	*p*
4	3.18 ± 0.169	2.85–3.51	1.06	0.291
5	3.61 ± 0.076	3.46–3.76	8.04	<0.001
6	2.79 ± 0.107	2.58–3.00	−1.97	0.052
7	2.97 ± 0.092	2.79–3.15	−0.32	0.748
8	2.91 ± 0.103	2.70–3.11	−0.92	0.359
9	2.90 ± 0.115	2.67–3.12	−0.89	0.374
10	3.29 ± 0.111	3.08–3.51	2.66	0.009
11	3.50 ± 0.167	3.18–3.83	3.02	0.003

**Table 5:** Parameter estimates of the final hurdle negative binomial model (interaction model).

Variable	Zero Hurdle Part (Infection Probability)			Count Part (Infection Intensity)		
	OR	95% CI	*p*	IRR	95% CI	*p*
Intercept	0.000	(0.000–0.043)	<0.05	13.656	(0.023–8155.642)	0.423
Length	1.916	(1.232–2.977)	<0.05	0.919	(0.533–1.586)	0.762
May	7.723	(0.013–4704.319)	0.532	0.000	(0.000–0.001)	<0.05
June	0.258	(0.000–608.614)	0.733	0.044	(0.000–820.008)	0.534
July	11.211	(0.018–7152.126)	0.463	0.246	(0.000–374,291.678)	0.847
August	3.850	(0.006–2514.482)	0.684	0.000	(0.000–1.666)	0.064
September	12.783	(0.012–14,039.640)	0.476	0.057	(0.000–2442.673)	0.598
October	140.656	(0.145–136,018.646)	0.158	0.002	(0.000–13.624)	0.163
November	4393.496	(9.136–2,112,741.343)	<0.05	0.025	(0.000–198.328)	0.420
Sex (female vs. male)	1.306	(0.859–1.985)	0.212	1.638	(0.894–3.003)	0.111
Sex (unknown vs. male)	0.609	(0.247–1.500)	0.281	1.752	(0.426–7.200)	0.437
Length × May	0.840	(0.477–1.481)	0.548	4.847	(1.612–14.577)	<0.05
Length × June	1.208	(0.582–2.508)	0.613	1.267	(0.510–3.147)	0.611
Length × July	0.804	(0.430–1.505)	0.496	0.718	(0.148–3.491)	0.681
Length × August	0.959	(0.524–1.757)	0.892	1.844	(0.802–4.243)	0.150
Length × September	0.790	(0.414–1.508)	0.474	0.972	(0.362–2.614)	0.956
Length × October	0.600	(0.315–1.143)	0.120	1.456	(0.633–3.350)	0.377
Length × November	0.418	(0.228–0.766)	<0.05	1.040	(0.414–2.608)	0.934
Log(theta)	–	–	–	0.424	(0.166–1.083)	0.073

Note: OR = odds ratio; IRR = incidence rate ratio.

## Data Availability

The data presented in this study are available upon request from the corresponding author due to privacy/ethical restrictions.

## Supplementary Materials

The following supporting information can be downloaded at: https://www.mdpi.com/article/10.3390/vetsci13070625/s1, Figure S1: Residuals vs. Fitted.

## Abbreviations

The following abbreviations are used in this manuscript:

ITS	Internal Transcribed Spacer
Krel	Relative Condition Factor
OR	Odds Ratio
IRR	Incidence Rate Ratio
AIC	Akaike Information Criterion
PCR	Polymerase Chain Reaction
NCBI	National Center for Biotechnology Information

## References

[1] Poulin R. (1999). The Functional Importance of Parasites in Animal Communities: Many Roles at Many Levels?. Int. J. Parasitol..

[2] Coen L.D., Bishop M.J. (2015). The Ecology, Evolution, Impacts and Management of Host–Parasite Interactions of Marine Molluscs. J. Invertebr. Pathol..

[3] Wood C.L., Byers J.E., Cottingham K.L., Altman I., Donahue M.J., Blakeslee A.M. (2007). Parasites Alter Community Structure. Proc. Natl. Acad. Sci. USA.

[4] Liu J., Xiao L., Zhang J., Wang B., Du M., Chen J., Zhu J., Shi L., Yang K. (2026). Ecological Patterns and Spatial Distribution of Medicinal Mollusks in a Freshwater Ecosystem. Parasites Vectors.

[5] Liu Y. (2024). Investigation on Wetland Resource Protection in Hami City. Xinjiang For..

[6] Zhou Z., Dang R., Yang S., Wang L., Xu J., Li J., An L. (2025). Spatiotemporal Variation Characteristics and Influencing Factors of Oases in Tuha Region over the Past 40 Years. Arid Land Geogr..

[7] Di Maggio M., Coltraro M., Tinacci L., Guardone L., Ricci E., Corradini C., Susini F., Armani A. (2024). Mapping the Occurrence of *Eustrongylides* spp. in Fish Species Caught from Six Lakes in Central Italy (Tuscany and Latium Regions): Implications for Local Fishery Supply Chains. Heliyon.

[8] Campião K.M., da Luz Rico J.A., de Souza Monteiro G., Ash L.V., Teixeira C.P., Gotelli N.J. (2024). High Prevalence and Concomitant Infection of *Ranavirus* and *Eustrongylides* sp. in the Invasive American Bullfrog in Brazil. Parasitol. Int..

[9] Fusco M., Rizzo-Valente V., Vizzoni V., Miranda R., Aguiar C., Escaleira R. (2023). An Outbreak of the Nematode Parasite *Eustrongylides* spp. (Nematoda: Dioctophymatidae) in a *Zebrafish* (Danio Rerio) Facility. Zebrafish.

[10] Xia S., Zhang L., Yang Y., Shi C., Wang J., Yue C., Wang X., Guo W., Hao C. (2025). Population Dynamics and Distribution Pattern of *Eustrongylides* sp. Parasitizing *Triplophysa microphthalma*. Acta Hydrobiol. Sin..

[11] Xia S., Sun H., Yang Y., Li Z., Ma Y., Liu F., Yang Y., Shi W., Yue C., Hao C. (2026). Morphological Redescription and Molecular Determination of *Eustrongylides tubifex* (Nematoda), A New Record Species in China. Acta Hydrobiol. Sin..

[12] Margolis L., Esch G.W., Holmes J.C., Kuris A.M., Et A. (1982). The Use of Ecological Terms in Parasitology (Report of an Ad Hoc Committee of the American Society of Parasitologists). J. Parasitol..

[13] Bush A., Lafferty K., Lotz J., Shostak A. (1997). Parasitology Meets Ecology on Its Own Terms: Margolis et al. Revisited. J. Parasitol..

[14] Gardes M., Bruns T.D. (1993). ITS Primers with Enhanced Specificity for Basidiomycetes—Application to the Identification of Mycorrhizae and Rusts. Mol. Ecol..

[15] Xu D., Li J., Wang J., Chen X., Song J., Wang X., Zhao G. (2025). Identification of *Eustrongylides* sp. from stomach of Nipponia nippon. Prog. Vet. Med..

[16] Yang R., Tian H., Zou L., Wang S., Li D., Li W. (2025). Polymorphism of the internal transcribed spacer sequences of *Eustrongylides* spp. in Monopterus albus. Acta Parasitol. Medica Entomol. Sin..

[17] Zhao D., Ye T., Gao F., Jakovlić I., La Q., Tong Y., Liu X., Song R., Liu F., Lian Z.-M. (2025). PhyloSuite v2: The Development of an All-in-One, Efficient and Visualization-Oriented Suite for Molecular Dating Analysis and Other Advanced Features. iMeta.

[18] Shamsi S., Francis N., Masiga J., Barton D., Zhu X., Pearce L., McLellan M. (2023). Occurrence and Characterisation of *Eustrongylides Species* in Australian Native Birds and Fish. Food Waterborne Parasitol..

[19] Kuraiem B., Verícimo M., Knoff M., Mattos D., São Clemente S. (2021). Sensitization with *Eustrongylides* sp. (Nematoda: Dioctophymatidae) Antigens Induce Production of Specific IgG and IgE in Murine Model. Rev. Bras. Parasitol. Vet..

[20] Zhang S., Huang G., Li L., Liu X., Tang X., Suo X. (2021). Morphological and Phylogenetic Analysis of *Eustrongylides* sp. and *Gnathostoma spinigerum* Parasitizing the Asian Swamp Eel Monopterusalbus in China. Pathogens.

[21] Franceschini R., Valiani A., Ranucci D., Roila R., Palma G., Agnetti F., Di Giacinto G., Branciari R. (2023). *Eustrongylides* spp. Parasite Risk Management in Atherina Boyeri from Lake Trasimeno. Ital. J. Food Saf..

[22] Kundu I., Bandyopadhyay P., Mandal D., Gürelli G. (2016). Study of Pathophysiological Effects of the Nematode Parasite *Eustrongylides* sp. on Freshwater Fish *Channa Punctatus* by Hematology, Serum Biochemical, and Histological Studies. Turk. Parazitolojii Derg..

[23] Nachev M., Schertzinger G., Sures B. (2013). Comparison of the Metal Accumulation Capacity between the Acanthocephalan *Pomphorhynchus Laevis* and Larval Nematodes of the Genus *Eustrongylides* sp. Infecting Barbel (*Barbus barbus*). Parasites Vectors.

[24] Morey G., Rojas C., Marin G., Guardia C. (2022). Occurrence of *Eustrongylides* sp. (Nematoda: Dioctophymatidae) in Fish Species Collected in the Peruvian Amazonia and Its Implications for Public Health. Acta Parasitol..

[25] Guagliardo S., Viozzi G., Brugni N. (2019). Pathology Associated with Larval *Eustrongylides* sp. (Nematoda: Dioctophymatoidea) Infection in *Galaxias Maculatus* (Actinopterygii: Galaxiidae) from Patagonia, Argentina. Int. J. Parasitol. Parasites Wildl..

[26] Moravec F., Nie P., Wang G. (2003). Some Nematodes of Fishes from Central China, with the Redescription of Procamallanus (Spirocamallanus)Fulvidraconis (Camallanidae). Folia Parasitol..

[27] Fayet A. (2020). Exploration and refinement of migratory routes in long-lived birds. J. Anim. Ecol..

[28] Tao K., He C., Zhang T., Xiao C., Du L., Li Z., Shao D., Wei J., Li B., Qiu Y. (2025). Evidence of WNV Infection in Migratory Birds Passing through Xinjiang, China, Using Viral Genome Amplicon Approach. Front. Microbiol..

[29] Chubb J. (1982). Seasonal Occurrence of Helminths in Freshwater Fishes. Part IV. Adult Cestoda, Nematoda and Acanthocephala. Adv. Parasitol..

[30] Altizer S., Dobson A., Hosseini P., Hudson P., Pascual M., Rohani P. (2006). Seasonality and the Dynamics of Infectious Diseases. Ecol. Lett..

[31] Turner W., Getz W. (2010). Seasonal and Demographic Factors Influencing Gastrointestinal Parasitism in Ungulates of Etosha National Park. J. Wildl. Dis..

[32] Marcogliese D. (2001). Implications of Climate Change for Parasitism of Animals in the Aquatic Environment. Can. J. Zool..

[33] Stromberg B. (1997). Environmental Factors Influencing Transmission. Vet. Parasitol..

[34] Fastzkie J., Crites J. (1977). A Redescription of *Eustrongylides Tubifex* (Nitzsch 1819) Jägerskiöld 1909 (Nematoda: Dioctophymatidae) from Mallards (Anas Platyrhynchos). J. Parasitol..

[35] Asakawa M., Kimoto Y., Murata K. (1997). First Record of *Eustrongylides tubifex* (Dioctophymatidae) from Little Grebe, Tachybaptus Ruficollis in Japan. J. Vet. Med. Sci..

[36] Shearer C., Ezenwa V. (2020). Rainfall as a Driver of Seasonality in Parasitism. Int. J. Parasitol. Parasites Wildl..

[37] Sures B., Nachev M. (2022). Effects of Multiple Stressors in Fish: How Parasites and Contaminants Interact. Parasitology.

[38] Zhao J., Qiu L., Zhou Q., Zhou X., Shen J. (2023). Reproductive strategy of Triplophysa yarkandensis in the Cherchen River, Xinjiang. J. Fish. Sci. China.

[39] Chaves-Pozo E., Liarte S., Fernández-Alacid L., Abellán E., Meseguer J., Mulero V., García-Ayala A. (2008). Pattern of Expression of Immune-Relevant Genes in the Gonad of a Teleost, the Gilthead Seabream (*Sparus aurata* L.). Mol. Immunol..

[40] Tomita Y., Kato K., Washio Y., Shirakashi S. (2024). Infections of Philometra Madai (Nematoda: Philometridae) in the Gonads of a High-Growth Broodstock Population of Red Seabream Pagrus Major. Fish Pathol..

[41] Nie P. (1990). A Review of Studies on Parasite Population Ecology. Acta Hydrobiol. Sin..

[42] Lafferty K.D., Kuris A.M. (1999). How Environmental Stress Affects the Impacts of Parasites. Limnol. Oceanogr..

[43] Kuraiem B., Knoff M., Telleria E., Fonseca M., Machado L., da Cunha N.C., Nascimento E.R.D., Fontenelle G., Gomes D.C., de São Clemente S.C. (2019). *Eustrongylides* sp. (Nematoda, Dioctophymatoidea) Parasitizing *Hoplias Malabaricus* (Actinopterygii: Erythrinidae) Collected from the State of Rio de Janeiro, Brazil. Rev. Bras. Parasitol. Vet..

[44] Melo F., Melo Cdo S., Nascimento Lde C., Giese E., Furtado A., Santos J. (2016). Morphological Characterization of *Eustrongylides* sp. Larvae (Nematoda, Dioctophymatoidea) Parasite of *Rhinella Marina* (Amphibia: Bufonidae) from Eastern Amazonia. Rev. Bras. Parasitol. Vet..

[45] Dezfuli B., Manera M., Lorenzoni M., Pironi F., Shinn A., Giari L. (2015). Histopathology and the Inflammatory Response of European Perch, *Perca Fluviatilis* Muscle Infected with *Eustrongylides* sp. (Nematoda). Parasites Vectors.

[46] Franceschini R., Guardone L., Armani A., Ranucci D., Roila R., Valiani A., Susini F., Branciari R. (2022). Five-Years Management of an Emerging Parasite Risk (*Eustrongylides* sp., Nematoda) in a Fishery Supply Chain Located on Trasimeno Lake (Italy). Food Control.

[47] Zaharieva R., Zaharieva P., Kirin D. (2023). Ecological Study on Helminths of Three Species of Gobiidae from the Danube River, Bulgaria. Helminthologia.

[48] Shen S. (1981). Discovery of *Eustrongylides mergorum* in China. Acta Zootaxonomica Sin..

