# Supplementary figures and images for: Epidemiology of Eustrongylides sp. Infection in Triplophysa strauchii: Temporal Dynamics and Risk Factors
Source: Vet Sci. 2026 Jun 26;13(7):625. doi: 10.3390/vetsci13070625 (PMC13431392; doi:10.3390/vetsci13070625)

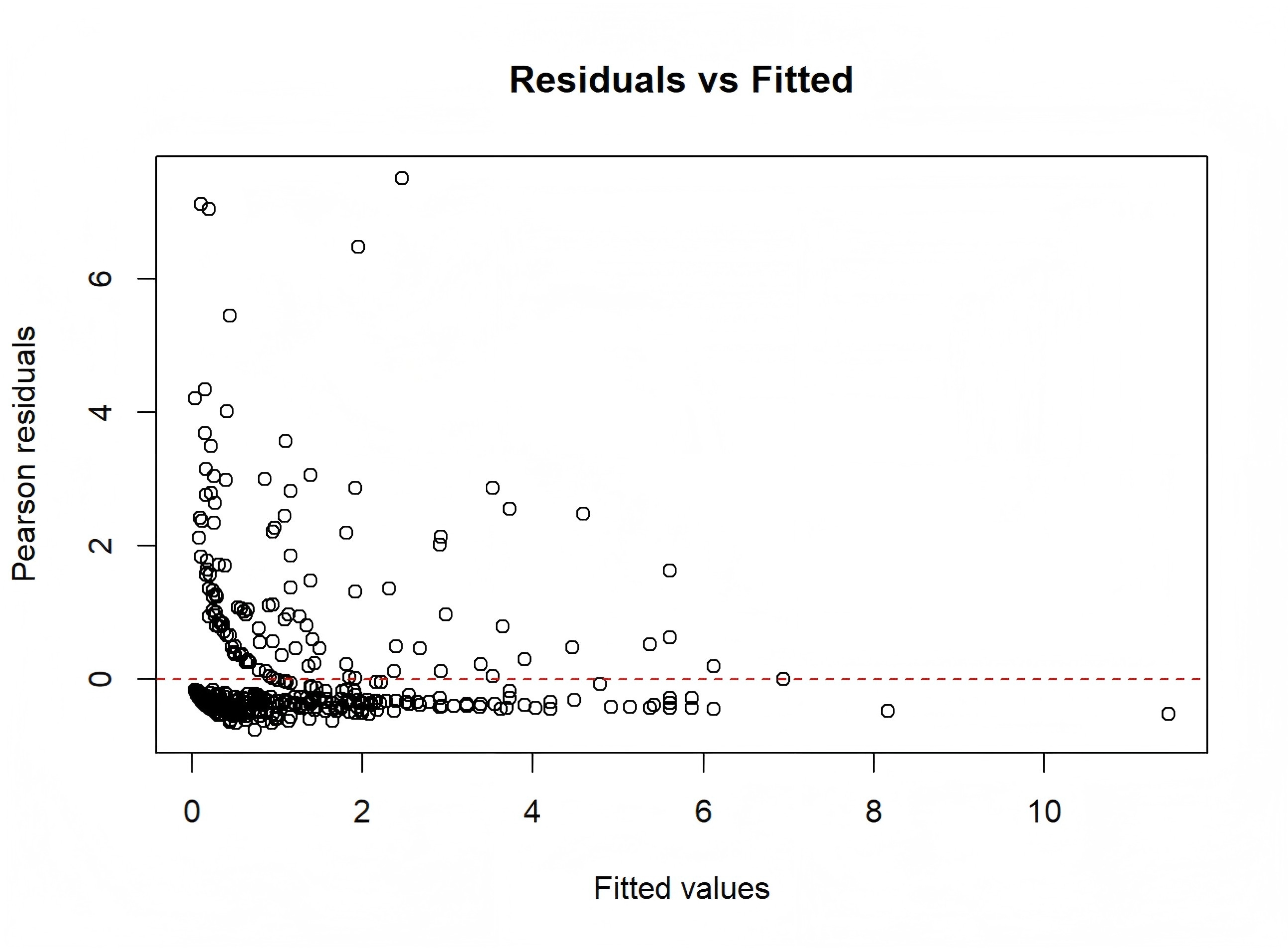

Supplement: Supplementary file 1 [file vetsci-13-00625-s001.zip › vetsci-4365115-supplementary.jpg]
